# Characterization of the Orange Juice Powder Co-Product for Its Valorization as a Food Ingredient

**DOI:** 10.3390/foods12010097

**Published:** 2022-12-24

**Authors:** Nuria Martínez-Navarrete, Eva García-Martínez, María del Mar Camacho

**Affiliations:** Food Technology Department, Food Investigation and Innovation Group, Universitat Politècnica de València, Camino de Vera s/n, 46022 Valencia, Spain

**Keywords:** upcycling, citrus industry waste, food ingredient powder, bioactive compounds, flowability, rehydration properties, techno-functional properties, gum Arabic, octenyl succinate anhydride starch, encapsulation

## Abstract

The citrus juice industry produces a large amount of fiber-rich waste and other bioactive compounds of great interest for their potential health benefits. This study focuses on the valorization of the co-product resulting from the extraction of orange juice to offer it as a versatile, healthy, high-quality, and stable natural food ingredient in powder form. To this end, the vitamin C (VC) content (ascorbic and dehydroascorbic acid, AA and DHAA), major flavonoids (hesperidin and narirutin, HES and NAT), and techno-functional properties (angle of repose, AoR; hygroscopicity and wettability; density and porosity; mean particle size, MPS; water retention capacity, WRC; oil holding capacity, ORC; emulsifying and foaming capacity, EC and FC; and emulsion and foam stability, ES and FS) have been characterized. In addition, considering that dehydrated foods with high sugar content require the incorporation of high molecular weight biopolymers for their physical stabilization, the influence of starch modified with octenyl succinic acid (OSA) and gum Arabic (GA) on these properties has been studied. The results obtained confirm the high quality of this co-product to be offered as a powdered food ingredient with nutraceutical potential. The addition of the studied biopolymers is recommended as it does not modify the flowability of the powder and favors both the encapsulation of the bioactive compounds, especially in the presence of GA, and the rehydration capacity.

## 1. Introduction

The sweet orange fruit is the most widely distributed species, with the highest production among all citrus species. The world orange production has reached 50 million tons. The main producer is Brazil (22.1%), followed by India (13.1%) and China (9.9%) [1]. In the last 25 years, the world orange production has been increasing (1.8 million tons more than the previous year 2021/22) in all countries except the USA, in which orange production has decreased [1]. The consumption of fresh oranges is the most common and recommended use. However, numerous companies are totally or partially involved in the transformation of oranges to juices or the preparation of preserves, forming a very dynamic sector, both in the national market and in the export market. This type of industry faces one of the main current problems of the food chain, which is the large amount of waste generated during the processing of the fruit, consisting mainly of the peel and cells of the fruit. The juice processing industry annually processes more than 27% of the world’s orange production, and approximately 50% of the total weight of the fruit becomes waste [1]. These residues are rich in macronutrients and other bioactive compounds of great interest for their potential health benefits [2,3,4]. Upcycling is one of the current trends to find solutions to deal with food waste, in line with what is being promoted by the European Union. It consists of using waste to create products with a higher value than the original material. In this way, instead of speaking about by-products or waste, they would acquire the co-product category. Citrus industry wastes have traditionally been used for animal feed and fertilizer production. However, other applications have been studied as bio-adsorbent material to capture heavy metals, as biofuel, or for the extraction of some bioactive compounds [5]. This last application, however, still generates a by-product.

Looking for alternative applications, the valorization of orange residue for its use as an ingredient in human food, in an integrated zero-waste process, could be a stimulating field of study to guarantee environmental protection, thereby promoting economic development and, at the same time, contributing to a sustainable and healthy diet. Owing to the high water content of the waste obtained after orange processing, it can deteriorate and reduce its shelf life. To solve this problem, drying processes could be carried out, which would also reduce the weight and volume of the obtained product, making it easier to handle. The dehydrated waste could be offered in powder form, a format in which many spices are already marketed. However, due to the high sugar content of the fruits, this powdered product is susceptible to water uptake from the environment relatively quickly and, therefore, to caking phenomena. To resolve this problem, high molecular weight solutes can be added to the product to improve its stability and facilitate its storage and marketing [6]. In this work, GA and OSA were used for this purpose.

OSA starch is obtained via an esterification reaction, resulting in a molecule of high molecular weight and amphiphilic nature. In the chemical modification of starch by OSA, hydrophilic and hydrophobic bifunctional groups are added to the molecule. OSA-modified starches have been used for different applications, particularly as food additives (E1450). Due to their branched structure and the hydrophobic and steric contribution of OSA, OSA-modified starches have interesting rheological and stabilizing properties, as well as encapsulating capacity. It is colorless and tasteless in solution, and its stabilizing capacity is independent of the pH and ionic strength of the medium [7]. GA is a natural-origin polysaccharide extracted from the resin of acacia trees. It is highly soluble in water and allows to obtain solutions in a wide range of concentrations without becoming excessively viscous. In addition, GA has properties as an emulsifier, stabilizer of dehydrated foods, thickener, and anti-caking agent, among others. An encapsulating effect that protects against oxidation and loss of volatile substances has also been described. Its use is widespread within the food and pharmaceutical sector [6,8]. In addition to the encapsulating role described for both biopolymers, their high molecular weight increases the glass transition temperature of the matrix to which they are added, which delays caking phenomena in powdered products [6,9,10]. However, their presence can affect some powder characteristics, an aspect that needs to be known.

This study focuses on the valorization of the waste from the extraction of orange juice to offer a versatile co-product, as a natural, healthy, high-quality, and stable powdered food ingredient, in an integrated zero-waste process. To ensure its success, different aspects of interest have been studied, such as its VC content (AA and DHAA), major flavonoids (HES and NAT), and techno-functional properties (AoR; hygroscopicity and wettability; density and porosity; MPS; WRC and OHC; and emulsifying and foaming capacity; ES and FS). In addition, the effect of GA and OSA incorporation on all the analyzed properties was studied.

## 2. Materials and Methods

### 2.1. Obtaining Powdered Co-Products under Study

The orange juice waste, consisting of both the peel and pulp discarded after juice extraction (Speed Up, Zumex, Valencia, Spain), was provided in October 2021 by the cafeteria of the Facultat de Belles Arts Sant Carles at the Universidad Politècnica de València (Spain). The waste, after cleaning with running water and cutting, was mixed with distilled water to obtain the CoP sample, or with a solution of OSA (Roquette, Lestrem, France) or GA (Scharlab, Valencia, Spain) in distilled water, prepared 24 h earlier, according to the percentages shown in Table 1, to obtain the co-product formulations OSA25, OSA45, and GA45. These formulations were prepared according to the recommendation of a study in which the formulation GA45 gave optimal results in relation to the properties of a freeze-dried orange puree powder [11]. However, considering the high fiber content of the waste used in this study, it was decided to avoid the incorporation of the minority amount of bamboo fiber according to the mentioned study and also test, with one of the two biopolymers, a lower concentration (0.25 g OSA/g CoP dry solutes). The mixtures were milled in batches of 750 g of waste for 5 min (Eurofred, Valencia, Spain), after which the mix obtained was distributed in aluminum trays of 25 cm in diameter, thickness 1 cm, and frozen at −45 °C (≥48 h, Liebherr LGT 2325, Baden-Wurtemberg, Germany) before being dried in a Telstar Lyoquest-55 freeze-drier (Telstar, Terrassa, Spain) operating at −50 °C in the condenser, 0.05 mbar and shelf temperature of 50 °C, for 18 h. The obtained product was ground in batches of 40 g for 20 s at speed 5 (Thermomix TM 21, Vorwerk, Valencia, Spain). A part of the powder was separated for particle size characterization and the rest was sieved using a 500 µm mesh (CISA, Barcelona, Spain) for 5 min at 50 Hz, amplitude of 2.5 mm (AMP0.40 CISA sieve shaker, Barcelona, Spain) for the analysis of the rest of the properties, as described below. The powder obtained was stored in zip bags under refrigeration, as to ensure the glassy state, thereby avoiding the caking phenomena [11], until its subsequent use in the corresponding tests.

### 2.2. Characterization of the Co-Product before Freeze-Drying and in Powdered Form

Each of the 4 considered co-product formulations was analyzed before and after freeze-drying to determine their water (x_w_), AA, DHAA, VC, HES, and NAT contents. Before freeze-drying, x_w_ was analyzed gravimetrically in a vacuum oven (Vaciotem, JP Selecta, Spain) and for the freeze-dried samples by using an automatic Karl Fischer titrator (C10S Mettler Toledo Compact Coulometric KF Titrator, Columbus, OH, USA). AA, VC, HES, and NAT were determined via UHPLC (Jasco equipment, Italy) using the methods proposed by Galindo et al. [12]. Briefly, to determine AA, an extraction with oxalic acid was performed and filtered through a 0.45 μm membrane filter before injection. The procedure employed to determine VC was the reduction DHAA, using DL-dithiothreitol as a reductant reagent. Then, for extraction, the same procedure was used, i.e., that used for the AA method. The UHPLC analysis was performed using a mobile phase of 0.1% oxalic acid and at a wavelength of 243 nm at 25 °C. AA standard solution (Dr. Ehrenstorfer, Germany) was prepared. The analysis of HES and NAT was carried out at a wavelength of 284 nm, using a gradient of water and methanol grade HPLC (VWR, Spain) for the mobile phase. HES and NAT were extracted with dimethyl-sulfoxide (Scharlau, Spain) grade HPLC under magnetic agitation for 10 min (MS-51M, Jeio Tech, Korea). Subsequently, the solution was centrifuged at 2031× *g* (Gyrozen 1236R, Daejeon, Korea) for 10 min at 4 °C. The supernatant was filtered through a 0.45 μm membrane filter for injection into the HPLC. The measurements were taken in triplicate. In addition, the four samples of the powdered co-product were characterized for the particle size distribution, flowability, rehydration capacity, and ability to interact with water or oil. All properties measured for this characterization, described below, were analyzed in triplicate, and the results are expressed as the mean value ± standard deviation.

For particle size distribution, 40 g of each sample was sieved using sieves of 800, 500, 300, 300, 200, 150, 100, 63, 45, 32, and 25 µm mesh size and bottom (5 min at 50 Hz, amplitude of 2.5 mm). From these distributions, the MPS, mode, and median were obtained for each sample. The powder flowability was evaluated from the AoR, porosity, and Hausner (HI) and Carr (CI) indexes. In all these cases, the methodology described by Uscanga et al. [13] was followed.

The properties selected to study the ability of the powders to interact with water were wettability, hygroscopicity, dispersibility, solubility, and WRC. Wettability is inversely proportional to the time required to wet all the powder particles when it is poured over water. To perform this analysis, 10 g of sample was poured over 250 mL of water at 25 °C. The time (s) in which all the particles completely sank and disappeared from the surface was recorded. Thus, the longer the wetting time, the lower the wettability of the sample. The hygroscopicity was obtained from the water gained by 1 g powder after exposure for 1.5 h in an environment with 75% relative humidity, created using a saturated NaCl solution. The result was expressed as g of water gained/100 g product dry solutes (ds). Dispersibility was determined as described by Jaya and Das [14], based on quantifying the dry solids from a powder in water solution that passes through a 200 µm sieve. Solubility was calculated as the percentage of soluble solutes present in a solution of 0.4 g powder in 12 mL of water, relative to total solutes [15]. For the determination of WRC, 0.5 g of the sample was vortexed in a 15 mL centrifuge tube (RA0015, Durviz, Valencia, Spain) with 4.5 mL of distilled water, allowed to stand for 18 h at 25 °C, and centrifuged at 1997 RCF and 20 °C for 25 min (Gyrozen 1236R, Daejeon, Korea). The supernatant was weighed before and after drying at a constant weight. The difference in weight made it possible to know the amount of water present, which referred to the mass of dry residue, thereby providing the WRC.

Regarding the ability of the powders to interact with oil, the OHC, EC, ES, FC, and FS were analyzed. To determine the OHC, the increase in weight of a mixture of 0.2 g of each powdered sample with 1.5 g of sunflower oil was recorded after centrifugation and resting [2]. For EC and ES, the methodology described by Yasumatsu et al. [16] was applied, with some modifications. A 10% (*w*/*v*) solution of powder in water was prepared and stirred on the magnetic stirrer (MS-51 M, Jeio Tech, Seoul, Korea) at 200 rpm for 5 min. After this, 7 mL of this solution and 7 mL of sunflower oil were mixed (Ultra-turrax Velp Scientifica ZX3, Usmate Velate, Italy) at 10,000 rpm for 1 min. Approximately 8 mL of this mixture (V) was centrifuged at 11,515 RCF for 5 min at 20 °C. As a result of this step, four phases could be separated: a precipitate, above it an aqueous phase, an upper fatty phase, and an intermediate one between the aqueous and the fatty phases, which would be the emulsion. The volume of the emulsion (Ve) was calculated from its height measured with a caliper, and the EC was calculated as shown in Equation (1).

(1) $$\mathrm{EC}\left(\%\right)=\frac{\mathrm{Ve}}{V}*100$$

To obtain ES, the same procedure was carried out as done for the EC, but after obtaining the mixtures with the ultra-turrax and introducing them into the centrifuge tubes, they were heated at 80 °C for 30 min, and subsequently, cooled with running water for 15 min and then centrifuged under the aforementioned conditions. The same Equation (1) was used to calculate the ES.

To determine FC (Equation (2)) and FS (Equation (3)), 2 g of each sample was mixed with 40 mL of distilled water and homogenized with the ultra-turrax for 5 min at 10,000 rpm. The volume before homogenization (V) and those of the foam newly obtained (V_0_), and after 30 s (V_30_) and 60 s (V_60_) were calculated from the corresponding height measured with a caliper.

FC = 100 * (V_30_ − V)/V (2)

FS = 100 * V_60_/V_0_ (3)

### 2.3. Statistical Analysis

Significant differences between the four co-product powder samples were tested by analysis of variance (simple ANOVA), with a confidence level of 95% (*p*-value < 0.05). Statgraphics Centurion version XVIII software (StatPoint Technologies, Inc., The Plains, Virginia) was used to this end.

## 3. Results and Discussion

### 3.1. Water and Bioactive Compound Content

Table 2 and Table 3 show the content of water and bioactive compounds analyzed in the different samples before and after freeze-drying, respectively. The water content of the orange juice waste used as raw material in this study was 78.8 ± 0.5 g water/100 g waste. Since all the formulations prepared contain added water and some of them also biopolymers, the x_w_ showed significant differences between the different co-products (*p* < 0.05, Table 2). Ensuring a low water content is essential for the stability of powdered samples during storage [17]. The x_w_ values of less than 7% for all freeze-dried co-products (Table 3) are below the critical water content that ensures the glassy state of the powder and so its stability [18]. Given the different contents in the waste that provides the bioactive compounds studied, in the added biopolymers, and in water (Table 1), to compare the composition of the different co-products both before and after freeze-drying, it was necessary to refer the results to a calculation basis that does not change between samples, which in this case are the solutes of the waste itself. For this purpose, the amount of biopolymer added in each case to the 750 g waste (Table 1), the water content of the waste used as raw material, and the water content of the formulated and freeze-dried samples (Table 2 and Table 3, respectively) were considered.

Considering bioactive compounds, owing to the amount in which they are found in orange juice waste and their potential interest at the physiological level, the content of HES, NAT, and VC was analyzed. Both HES and NAT are the dominant flavonoids in citrus fruits, with HES being the majority in the peel [19]. Flavonoids may play a role as antioxidants; antimicrobial, anti-inflammatory, and antimutagenic compounds; and those that prevent different coronary diseases [20]. VC is found in two forms, AA and the oxidized DHAA. This last compound is predominant in orange peels, contrary to what occurs in the pulp [12]. In this case, its effect on vascular diseases, cancer, cataract prevention, and hypertension control has been described [19].

**Figure 1:**
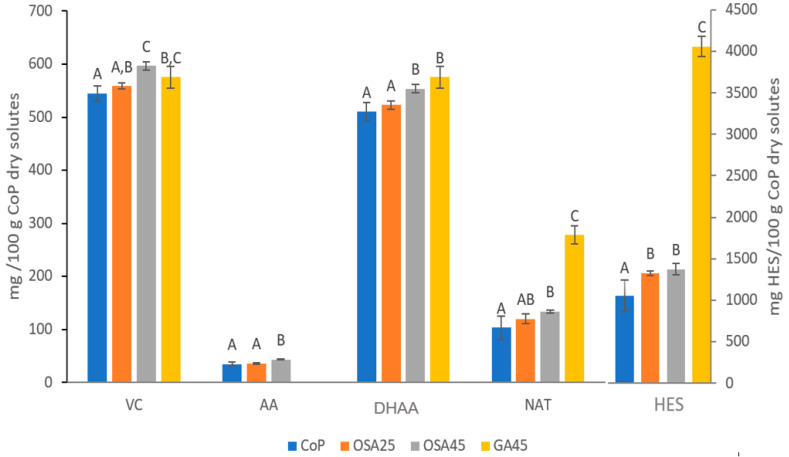
Bioactive compound content of the samples before freeze-drying (mg/100 g CoP dry solutes). VC: vitamin C; AA: ascorbic acid; DHAA: dehydroascorbic acid; NAT: narirutin; HES: hesperidin. For each com-pound, different letters (A–C) indicate different homogeneous groups established by Tukey HSD ANOVA (*p* < 0.05).

**Figure 2:**
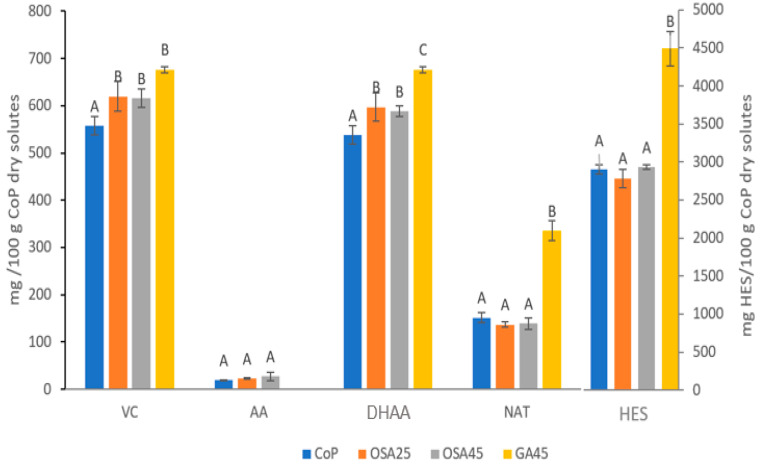
Bioactive compound content of the freeze-dried samples (mg/100 g CoP dry solutes). VC: vitamin C; AA: ascorbic acid; DHAA: dehydroascorbic acid; NAT: narirutin; HES: hesperidin. For each com-pound, different letters (A–C) indicate different homogeneous groups established by Tukey HSD ANOVA (*p* < 0.05).

In the samples studied, HES was the major component both before (Table 2, Figure 1) and after (Table 3, Figure 2) freeze-drying. In the first case, both HES and NAT concentrations increased with the addition of the biopolymers (*p* < 0.05, Figure 1). GA and OSA favor the extraction of bioactive compounds because they decrease the viscosity of the samples, which in turn favors encapsulation [12]. In the current case, this effect was more pronounced in the presence of GA than in the presence of OSA. In contrast, a higher concentration of added OSA was not more effective for the encapsulation of these compounds (*p* > 0.05). The HES and NAT content increased in the freeze-dried samples (Figure 1 and Figure 2), which may be due to the greater ease of extraction in this case, associated with the high porosity of the freeze-dried co-products [12].

As expected for VC, the DHAA content was higher than the AA content in all the samples (Figure 1 and Figure 2). The co-products with added biopolymers showed a higher VC concentration (Figure 1 and Figure 2). VC also showed a certain greater ease of extraction in the freeze-dried powders, which was a consequence of an increase in DHAA, significant for the samples with added biopolymers (*p* < 0.05), rather than the loss of AA (*p* < 0.05). Galindo et al. [12] justify this behavior based on the temperature used during freeze-drying (50 °C), which favors the degradation of AA to DHAA and the encapsulation of DHAA.

When the freeze-dried samples are compared, it can be observed that GA45 was the one that presented with the highest content in HES, NAT, DHAA, and VC (*p* < 0.05, Figure 2). In the case of this sample, an anomalous absence of AA was observed, which can be attributed to a problem in its handling. In contrast, if the different freeze-dried co-products are compared with the waste used as raw material, an increase in VC was observed, always at the expense of the increase in DHAA and decrease in AA. This increase was 2%, 13%, and 24% for the samples without biopolymers, those with OSA, and those with GA, respectively. The increase in HES was in the order of 172% for the samples without biopolymers and those with OSA and up to 325% for GA45. For NAT, these increases were 47%, 34%, and 226% for the samples without biopolymers, those with OSA, and those with GA, respectively. These results indicate the potential of GA for the encapsulation of the studied bioactive compounds by freeze-drying.

### 3.2. Techno-Functional Properties of Powdered Co-Products

**Figure 3:**
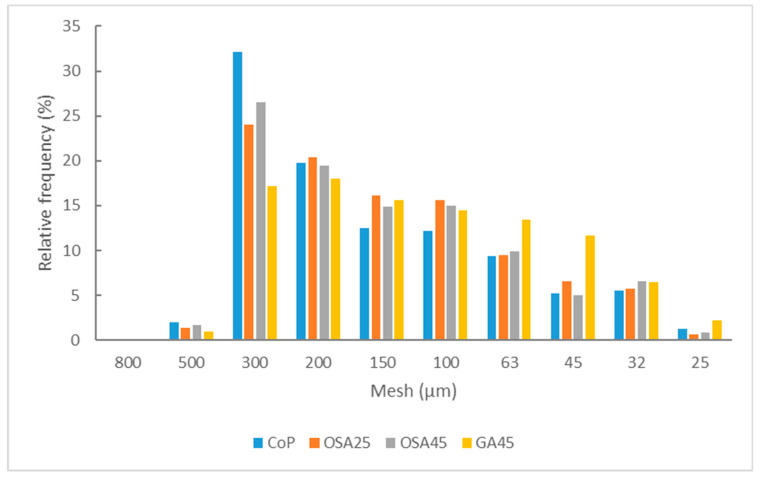
Particle size distribution of the different powder samples (codes according to Table 1) in relation to the percentage of sample retained in each of the sieves (relative frequency, %).

One approach to determine the resistance of freeze-dried cakes to crushing is based on the particle size distribution of the powder and the MPS, mode, and median obtained from it (Figure 3, Table 4). For this purpose, the particle size was the mean value between the mesh size of the sieve where the particles were retained and that of the sieve of the next larger size. As can be observed in Figure 3, the less homogeneous distribution was that of CoP, with more particles of larger size, and the most homogeneous was that of GA45. This makes the MPS oscillate between that of these two samples, 259 and 183 µm, respectively (*p* < 0.05). The size of the samples with OSA did not show significant differences with that of samples with CoP (Table 4, *p* > 0.05). Regarding the mode of the particle size distributions (Table 4), the sieve that retained the most particles was the 300 µm sieve, except in the case of GA45, for which it was the 200 µm sieve. Moreover, the median was lower for the sample with GA than for those with OSA, and the median of the latter was lower than that of those with CoP (Table 4, *p* < 0.05). This behavior may be related to a lower resistance to crushing of the cake obtained after freeze-drying the co-product containing GA.

Table 5 shows the physical properties related to the flowability measured for the powder co-products studied, related to the friction between the particles and their adhesiveness. When the flowability increases, the AoR, HI, and CI decrease. In this study, none of the three properties showed significant differences between samples (*p* > 0.05, Table 5). Although some studies describe that a smaller particle size means a worse flow of the particles of a powder [21], in this case, the smaller size of sample GA45 was not enough to decrease its flowability. The AoR of the powdered co-products (between 36.6 and 38, 4°), the HI (1.057–1.08), and the CI (5.4–7.1%) were of the same order or slightly lower than those found for a powder obtained from orange puree with different biopolymers added [10,22]. The lower fiber content and higher sugar content of the puree justify these results [23].

There are different classifications to characterize a powder based on its AoR. According to Barbosa-Canovas et al. [24], a material flows well when AoR < 35°, it is fairly cohesive if the value is 35–45°, it is cohesive if the AoR is 45–55°, and it is very cohesive if the AoR > 55°. As stated by Alavi and Caussat [25], the classification is somewhat different, so that high flowability is assumed if 25° < AoR < 30°, medium if 30° < AoR < 38°, and low when 38° < AoR < 45°. According to the RFE [26], good flowability is indicated when AoR is 31–35°, regular flowability when AoR is 36–40°, and acceptable when AoR is 41–45°. Powders with HI <1.25 and IC 5–15% are considered to have intermediate/high flow or excellent flow, respectively [27]. According to these classifications, the four samples studied would have to be considered somewhat cohesive, but with a more than acceptable flowability. This certain cohesiveness was to be expected considering the composition of these samples, with a relatively high sugar content. Thus, it is important to note that the presence of biopolymers does not improve the flowability of the co-product. If we take the powdered orange puree or juice as a reference, they were so cohesive that it is not possible to characterize their physical properties (unpublished information). In these cases, it was necessary to add biopolymers, in a comparable amount to that present in the OSA45 and GA45 samples, to obtain a powder with a flowability similar to that obtained in this study. Therefore, it seems that the higher and lower content of fiber and sugars, respectively, in the co-product than in the edible part of the fruit prevents the formation of interparticle bridges, ensuring a free-flowing powder, without the need to add OSA or GA.

To calculate the real density of the freeze-dried samples (Table 5), the amount of biopolymers added (Table 1) and the water content of the powders (Table 3) were considered, as well as the composition of the orange juice co-product [23]. With this data, the composition of each powdered product was calculated (Table 6), and considering the density of the pure components at 20 °C [28], Equation (4) was applied to obtain the real density. The higher carbohydrate content and lower water content of the samples with biopolymers justify their higher density. The bulk density of the tapped and poured powder without added biopolymers were lower than those of the formulated powders, with no significant differences between the latter (*p* > 0.05, Table 5). These properties help to know the ease with which a material can be packaged or mixed in a container. From this point of view, the incorporation of biopolymers implies a better packing of the particles and, therefore, a higher bulk density and lower interparticle porosity (Table 5).

Table 7 shows the value of the properties related to the interaction with water. Hygroscopicity refers to the ability of a product to absorb water from an environment with high relative humidity, and a good powdered product is one with low hygroscopicity [17]. In this case, the four samples studied showed values between 3.8 and 4.9 g water gained/100 g solutes, with no significant differences among them (*p* < 0.05). The hygroscopicity of the co-products was lower than that of other plant-derived fibers (14–35%, [29,30]), which can be justified by the presence of other components that have less affinity to water in the samples characterized in this study. The low hygroscopicity of the powder co-product will help it to remain free-flowing during storage. This, together with the fact that the presence of the added biopolymers does not modify the tendency of the powdered co-product to take up water from the environment indicates that the incorporation of biopolymers into the co-product is not necessary from the point of view of the physical stability of the powder and to prevent caking phenomena.

Wettability along with dispersibility and solubility are among the most important properties in powdered products that have to be rehydrated for use [15]. In this study, the samples showed a significant increase in wettability (decrease of wetting time) in the presence of the biopolymers, especially OSA (*p* < 0.05, Table 7). The high insoluble fiber content and the presence of lipids in the co-product [18] along with the amphiphilic properties of GA [31] and OSA [32] justify this behavior. The higher interparticle porosity of the CoP sample may also contribute in this regard [33]. Dispersibility was also greatly enhanced by the presence of biopolymers, more so for GA than for OSA (*p* < 0.05, Table 7). The solubility of the co-product agrees with the value found by de-Moraes et al. [34]. It increased in the presence of a higher amount of added biopolymers (*p* < 0.05, Table 7), which may be related to the solubility of the biopolymers themselves. In contrast, the WRC was of the same order for all samples (Table 7), and lower than that of other fibers of vegetable origin (5–26% ds [29,30]), including that extracted from the by-product of the orange processing industry (6–10% ds [35,36]). Since this property is associated with the presence of fiber [35], the content of solutes other than fiber in the co-products under study may justify the lower value obtained.

Table 8 shows the results of the properties of the powdered samples related to their ability to interact with oil and incorporate air. The results of the OHC showed significant differences between CoP and the samples with added biopolymers. The samples with OSA had the lowest OHC, although without significant difference with GA45. The OHC of CoP was of the order of that found by de-Moraes et al. [34] and slightly higher than that of different plant-derived fibers (14–35% [29,30]). A high OHC can contribute to preventing fat loss during the cooking of food, in addition to lowering blood cholesterol levels [34]. In terms of EC and ES, no significant differences were observed between CoP and GA45, but they were lower than those of the sample with OSA (*p* < 0.05). This can be related to the amphiphilic character of OSA, due to the presence of hydrophilic and hydrophobic bifunctional groups, which favors the formation and stability of the emulsion [32]. Similar behavior was observed for FC, which was also higher in the samples with OSA, especially sample OSA45 (*p* < 0.05). However, OSA 45 sample showed the least stable foam (Table 8).

## 4. Conclusions

The incorporation of GA to the waste of orange juice favors the encapsulation of its VC, HES, and NAT by freeze-drying. OSA only showed an encapsulating effect on VC, which was lower than that of GA. In addition, GA seems to confer to less resistance to crushing to the freeze-dried co-product cakes, which facilitates obtaining a powder with a smaller and more homogeneous particle size. In contrast, neither GA nor OSA seem to be necessary to prevent caking phenomena, as they do not modify the flowability of the powders but they do improve their packaging. Regarding the use of the powdered co-product as an ingredient in the formulation of other foods, the presence of GA or OSA is desirable when rehydration is required before its final use since they reduce wetting time and increase dispersibility and solubility. However, they decrease the oil retention capacity of the powder, so they would not be suitable to formulate fatty foods. For its part, OSA promotes emulsifying properties as well as the foaming capacity. Based on the above, and considering the versatility of the powdered co-product, adding GA to the orange juice co-product before freeze-drying may be recommended, which will favor the presence of bioactive compounds and its rehydration capacity. This would contribute to the circular economy of the agri-food sector and thus reduce the environmental impact generated by these by-products. In addition, the results of this study allow us to recommend how to process it to ensure not only its stability but also a high content of bioactive compounds. From this point of view, the use of this powdered co-product as a food ingredient with nutraceutical potential may be proposed.

## Figures and Tables

**Table 1:** Orange juice co-product formulations without biopolymers added (CoP) or with 0.25 g OSA, 0.45 g OSA, or 0.45 g GA for each g of CoP dry solutes (OSA25, OSA45, and GA45, respectively).

Sample	Waste (g)	GA/OSA (g)	H_2_0 (mL) ^1^	H_2_0 (mL) ^2^
CoP	100	-	-	37.8
OSA25	100	7	21	16.8
OSA45	100	12.6	37.8	-
GA45	100	12.6	37.8	-

^1^ Volume of water in which the biopolymer is diluted for the incorporation into the formulation. ^2^ Volume of water added to the formulation.

**Table 2:** Mean water (x_w_, g water/100 g analyzed sample) and bioactive compound content (mg/100 g analyzed sample) of the different formulations before freeze-drying (codes according to Table 1): vitamin C (VC), ascorbic acid (AA), dehydroascorbic acid (DHAA), hesperidin (HES), and narirutin (NAT).

	CoP	OSA25	OSA45	GA45
x_w_	84.14 ± 0.03 ^c^	80 ± 2 ^b^	77.98 ± 0.02 ^a^	77.70 ± 0.02 ^a^
VC	86 ± 2 ^b^	82.1 ± 0.9 ^a^	82.3 ± 1.1 ^a^	80 ± 3 ^a^
AA	5.5 ± 0.6 ^a^	5.3 ± 0.2 ^a^	5.91 ± 0.08 ^a^	nd
ADHA	81 ± 3 ^b^	76.7 ± 1.1 ^a^	76.3 ± 1.1 ^a^	80 ± 3 ^a^
HES	167 ± 30 ^a^	195 ± 4 ^a^	189 ± 10 ^a^	567 ± 17 ^b^
NAT	16 ± 3 ^a^	17.6 ± 1.3 ^a^	18.5 ± 0.4 ^a^	39 ± 2 ^b^

Results expressed as mean ± standard deviation; nd: not detected. Different letters in superscripts (^a–c^) indicate significant differences among samples for each property (*p* < 0.05).

**Table 3:** Mean water (x_w_, g water/100 g analyzed sample) and bioactive compound content (mg/100 g analyzed sample) of the different freeze-dried formulations (codes according to Table 1): vitamin C (VC), ascorbic acid (AA), dehydroascorbic acid (DHAA), hesperidin (HES), and narirutin (NAT).

	CoP	OSA25	OSA45	GA45
x_w_	4.4 ± 0.3 ^b^	3.43 ± 0.08 ^a^	2.9 ± 0.3 ^a^	2.9 ± 0.3 ^a^
VC	526 ± 20 ^d^	449 ± 23 ^c^	374 ± 12 ^a^	411 ± 4 ^b^
AA	18.37 ± 0.09 ^a^	16.4 ± 1.1 ^a^	17 ± 6 ^a^	nd
DHAA	514 ± 19 ^c^	433 ± 22 ^b^	358 ± 7 ^a^	411 ± 4 ^b^
HES	2776 ± 56 ^c^	2018 ± 90 ^b^	1782 ± 17 ^a^	2728 ± 139 ^c^
NAT	135 ± 10 ^b^	99 ± 4 ^a^	85 ± 8 ^a^	204 ± 13 ^c^

Results expressed as mean ± standard deviation; nd: not detected. Different letters in superscripts (^a–c^) indicate significant differences among samples for each property (*p* < 0.05).

**Table 4:** Mean values of mean particle size (MPS), mode, and median (µm) of the different powder samples (codes according to Table 1).

	CoP	OSA25	OSA45	GA45
MPS	259 ± 9 ^b^	217 ± 3 ^b^	224 ± 3 ^b^	185 ± 3 ^a^
Mode	400 ^b^	400 ^b^	400 ^b^	250 ^a^
Median	303 ± 20 ^c^	242 ± 6 ^b^	255 ± 7 ^b^	209 ± 5 ^a^

Results are expressed as mean ± standard deviation. Different letters in superscripts (^a–c^) indicate significant differences among samples for each property (*p* < 0.05).

**Table 5:** Mean values of angle of repose (AoR), Hausner index (HI), Carr index (CI), real density (ρ), bulk density of tapped and poured powder (ρ_t_; ρ_p_), and porosity of tapped and poured powder (ε_t_; ε_p_) of the different powder samples (codes according to Table 1).

	CoP	OSA25	OSA45	GA45
AoR (°)	37 ± 3 ^a^	38.4 ± 0.2 ^a^	38.2 ± 1.1 ^a^	38.3 ± 0.4 ^a^
HI	1.057 ± 0.007 ^a^	1.057 ± 0.005 ^a^	1.08 ± 0.04 ^a^	1.07 ± 0.03 ^a^
CI (%)	5.4 ± 0.6 ^a^	5.4 ± 0.4 ^a^	7.1 ± 1.4 ^a^	7 ± 2 ^a^
ρ (g/cm^3^)	1.399	1.405	1.408	1.408
ρ_t_ (g/cm^3^)	0.144 ± 0.008 ^a^	0.237 ± 0.007 ^b^	0.31 ± 0.08 ^b^	0.281 ± 0.019 ^b^
ρ_p_ (g/cm^3^)	0.136 ± 0.006 ^a^	0.224 ± 0.006 ^b^	0.29 ± 0.07 ^b^	0.262 ± 0.016 ^b^
ε_t_ (%)	89.7 ± 0.6 ^b^	83.1 ± 0.5 ^a^	80 ± 5 ^a^	81.4 ± 1.2 ^a^
ε_p_ (%)	90.3 ± 0.5 ^b^	84.1 ± 0.4 ^a^	81 ± 5 ^a^	80.1 ± 1.2 ^a^

Results are expressed as mean ± standard deviation. Different letters in superscripts (^a–b^) indicate significant differences among samples for each property (*p* < 0.05).

**Table 6:** Composition (g/100 g) of the different formulations of the freeze-dried orange juice co-product (codes according to Table 1).

	CoP	OSA25	OSA45	GA45
Water	4.44	3.43	2.96	2.96
Carbohydrates	84.12	87.46	89.21	89.21
Proteins	6.24	4.97	4.27	4.27
Lipids	0.26	0.21	0.18	0.18
Ash	4.4	3.93	3.38	3.38

**Table 7:** Hygroscopicity (g water gained/100 g dry solutes), wettability (s), dispersibility (g dry solutes passing through the sieve/g dry solutes sample), solubility (%), and water retention capacity (WRC, %) of the different powder samples (codes according to Table 1).

	CoP	OSA25	OSA45	GA45
Hygroscopicity	4.1 ± 0.7 ^a^	4.17 ± 0.16 ^a^	3.8 ± 0.8 ^a^	4.9 ± 0.7 ^a^
Wettability	1845 ± 59 ^d^	269 ± 25 ^a^	412 ± 62 ^b^	598 ± 19 ^c^
Dispersibility	8 ± 2 ^a^	87 ± 15 ^b^	103 ± 5 ^b^	241 ± 20 ^c^
Solubility	29 ± 1 ^a^	32 ± 2 ^a^	45 ± 3 ^b^	45 ± 5 ^b^
WRC	2.33 ± 0.04 ^ab^	2.44 ± 0.03 ^b^	2.27 ± 0.11 ^a^	2.28 ± 0.05 ^a^

Results are expressed as mean ± standard deviation. Different letters in superscripts (^a–c^) indicate significant differences among samples for each property (*p* < 0.05).

**Table 8:** Water and oil retention capacity (WRC; OHC), emulsifying capacity and emulsion stability (EC; EE), and foaming capacity and stability (FC, FS) of the different formulations (codes according to Table 1).

	CoP	OSA25	OSA45	GA45
ORC (%)	2.69 ± 0.18 ^b^	1.9 ± 0.3 ^a^	1.77 ± 0.04 ^a^	2.06 ± 0.19 ^a^
EA (%)	8 ± 1 ^a^	29 ± 4 ^b^	30 ± 8 ^b^	7.8 ± 0.6 ^a^
EE (%)	8 ± 1 ^a^	24 ± 2 ^b^	24.3 ± 0.3 ^b^	7.0 ± 0.4 ^a^
FC (%)	3 ± 2 ^a^	9 ± 2 ^b^	20.1 ± 1.2 ^c^	4.88 ± 0.09 ^a^
FS (%)	95.08 ± 1.07 ^b^	95.2 ± 1.5 ^b^	85 ± 3 ^a^	93 ± 3 ^b^

Results are expressed as mean ± standard deviation. Different letters in superscripts (^a–c^) indicate significant differences among samples for each property (*p* < 0.05).

## Data Availability

Data is contained within the article.
